# Artificial intelligence fails to outperform orthopaedic surgeons: A systematic review

**DOI:** 10.1002/jeo2.70548

**Published:** 2025-11-14

**Authors:** Jemima Russell, Jamie Rosen, Martinique Vella‐Baldacchino

**Affiliations:** ^1^ Department of Surgery and Cancer MSk Lab—Imperial College London London UK; ^2^ Warwick Medical School University of Warwick Coventry UK

**Keywords:** artificial intelligence, ChatGPT, machine learning, orthopaedic surgery, orthopaedics

## Abstract

**Purpose:**

Artificial intelligence (AI) in orthopaedic surgery is increasingly applied to analyse clinical data, triage patients and interpret imaging with high accuracy. Orthopaedics surgery faces unique challenges, including high patient volumes, complex cases and prolonged waiting lists, highlighting the need for efficiency and decision support. To justify implementation, AI must demonstrate performance comparable to surgeons. This systematic review evaluates AI's performance relative to surgeons to determine its value as a complementary tool in orthopaedic practice.

**Methods:**

This systematic review was conducted using OVID Medline. Relevant studies published up to 13 August 2025 were identified. Included studies were categorised into decision making, management plans, clinical knowledge, quality control, and answering patients' frequently asked questions (FAQs).

**Results:**

Of 419 identified studies, 16 were eligible. ChatGPT showed high sensitivity in identifying patients achieving clinically meaningful improvements (97% vs. 90% for surgeons) but lower specificity (33% vs. 63%) and accuracy (65% vs. 76%). AI demonstrates comparable or superior performance to surgeons in emergency scenarios and answering patient FAQs, scoring higher across empathy, accuracy, completeness and overall quality (4.4 vs. 3.5–3.7). Residents outperformed AI in examinations (74.2% vs. 47.2%). AI showed limited accuracy in knee osteoarthritis radiographic staging (35% vs. >80%).

**Conclusions:**

AI demonstrates the potential to support clinical efficiency and patient communication in orthopaedics. However, concerns about bias, quality risks, overconfidence and reliance on outdated information prevent it from replacing human expertise. Clinician‐led design and validation are required to ensure safe and effective integration into clinical practice.

**Level of Evidence:**

Level IV.

## Graphical abstract

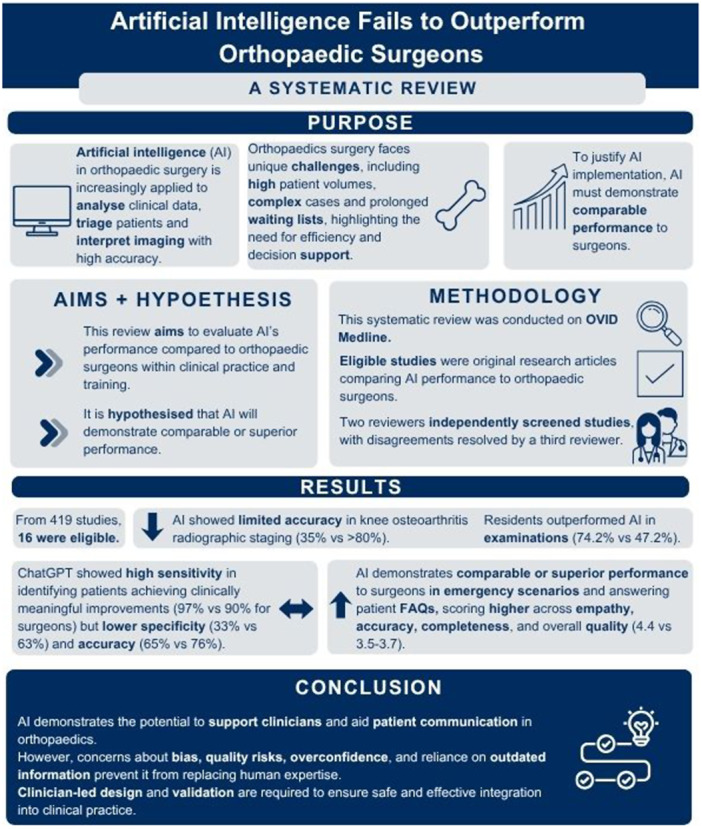

**Abbreviations**
AAOS: American Academy of Orthopaedic Surgeon
AI: artificial intelligence
ChatGPT: Chat Generative Pre‐Trained Transformer
EHR: electronic health record
FAQs: frequently asked questions
KOOS‐JR: Knee Osteoarthritis Outcome Score‐Joint Replacement
LLM: large language models
MCID: minimum clinically important difference
NLP: natural language processing
OITE: Orthopaedic In‐Training Examination
PGY: postgraduate year
PJI: periprosthetic joint infection
SSI: surgical site infection
TKR: total knee replacement
UKR: unilateral knee replacement

## INTRODUCTION

Artificial intelligence (AI) refers to the ability of machines to simulate human intelligence and perform tasks autonomously [29]. Large language models (LLMs), a type of conversational AI trained on hundreds of billions of textual data, have been developed for commercial use to engage in natural, contextually relevant conversations [16, 40]. Examples include Chat Generative Pre‐Trained Transformer (ChatGPT), Google Bard and Microsoft CoPilot [30, 39, 40].

Within healthcare, orthopaedic surgery faces unique challenges, including high case load, complex patient presentation and management [15]. Orthopaedics had the longest NHS waiting lists in 2022, with over 800,000 patients, half of whom waited longer than the NHS target of 18 weeks [47]. Addressing this backlog and streamlining future systems requires clinical pathways to help use healthcare resources more efficiently to improve patient care with AI as a potential to aid this [18].

AI plays an expanding role in healthcare to improve patient outcomes and alleviate the workload of healthcare professionals [6]. Most orthopaedic surgeons expect AI to substantially influence their field within 5–10 years [41]. Natural language processing (NLP) is trained to process and understand human language, allowing AI to answer frequently asked questions (FAQs) about a wide range of orthopaedic conditions accurately and triage patients based on reported symptoms [12, 43, 48]. Machine learning models use pattern recognition to analyse clinical records, genetic information and biomarkers to identify risk factors for conditions such as femoroacetabular impingement syndrome and periprosthetic joint infections (PJI) [12, 13, 22, 46]. AI enhances efficiency by optimising scheduling, reducing wait times and interpreting imaging studies, such as magnetic resonance imaging (MRI), X‐ray or computed tomography (CT) scans with high accuracy [12].

Notably, 91.1% of orthopaedic surgeons expect AI to act as a complementary tool, rather than a replacement [41]. To provide meaningful value, AI must perform at or above current surgeon‐level standards. Previous literature emphasises the need to transform theoretical concepts into practical clinical implementation [6]. Empirical data studies comparing AI to orthopaedic surgeons are essential to assess its real‐world value. This review aims to evaluate AI's performance relative to orthopaedic surgeons within clinical practice and training. It is hypothesised that AI will demonstrate comparable or superior performance, supporting its role as a potentially valuable complementary tool in clinical practice.

## METHODS

### Search strategy and study selection

**Figure 1:**
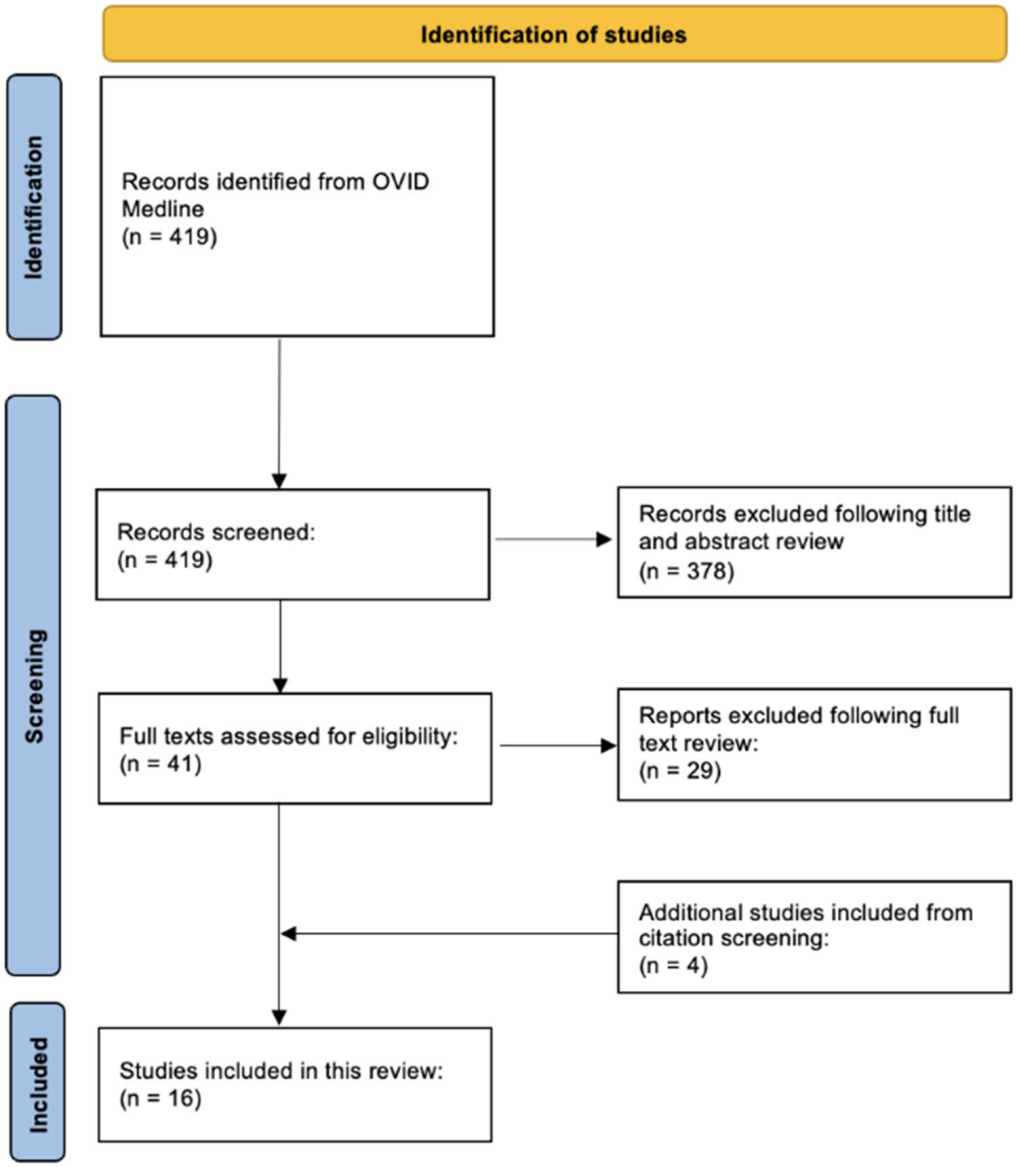
Preferred Reporting Items for Systematic Reviews and Meta‐Analyses (PRISMA) flow diagram illustrating study selection process, including the number of records identified, screened, excluded and included in the final review.

A systematic search was conducted using OVID Medline for studies published between the start of the database and 13 August 2025. The search terms are listed in Supporting Information S1: Appendix. Eligible studies were original research articles published in English evaluating AI in orthopaedic surgery and directly compared AI performance to orthopaedic surgeons. Studies were excluded if they only described theoretical AI models without empirical data, or compared AI to other healthcare professionals, for example, radiologists, orthopaedic specialist nurses or nonspecialist doctors. Systematic reviews were screened for relevant citations. Study selection is summarised in Figure 1.

The primary outcome was AI performance compared to orthopaedic surgeons, categorised into decision making, management planning, clinical knowledge, quality control and answering FAQs. Risk of bias was assessed using ROBINS‐I for non‐randomised studies, RoB2.0 for randomised controlled trials and QUADAS‐2 for diagnostic studies.

### Screening and data extraction

Two reviewers (J. Ru. and J. Ro.) independently scanned titles and abstracts for relevance using Covidence, and full texts were evaluated against the eligibility criteria. Disagreements between the reviewers were resolved with the involvement of a third reviewer (Martinique Vella‐Baldacchino). Reference lists of all the studies identified in the above methods were screened for additional studies of possible relevance. Two authors (J. Ru. and J. Ro.) independently extracted data using a standardised data‐extracting form, which included study location, AI model, surgeon experience and comparative performance, with any differences resolved by discussion.

## RESULTS

The search produced 419 eligible studies, of which 378 were excluded based on title/abstract screening and 29 through full‐text screening, with 4 identified by citation screening. A total of 16 studies were included, categorised as follows: decision making (*n* = 5), management plans (*n* = 2), clinical knowledge (*n* = 6), quality control (*n* = 1) and answering patient FAQs (*n* = 2) (Table 1).

**Table 1:** Summary of included studies.

Category	Study	Continent	Evidence level	Clinical focus
Decision making				
	Chester et al. [7]	Oceania	III	Surgical Site Infection in Paediatric Spine Surgery
	Zalikha et al. [51]	North America	V	Total Knee Arthroplasty
	Musbahi et al. [31]	Europe	III	Knee Replacement
	Liu et al. [24]	North America	III	Ankle Fractures
				Distal Radius Fractures
				Knee Septic Arthritis
				Shoulder Dislocations
				Achilles Tendon Ruptures
	Zhu et al. [53]	Asia	III	Knee Osteoarthritis
Management plans				
	Dagher et al. [9]	North America	III	Carpal Tunnel Syndrome
				Distal Radius Fracture
				Glenohumeral Joint Osteoarthritis
				Rotator Cuff Injury
				Clavicle Fracture
				Hip Fracture
				Hip Osteoarthritis
				Knee Osteoarthritis
				ACL Injury
				Achilles Rupture
	McNamara et al. [28]	North America	V	Fractures
Clinical knowledge				
	Yağar et al. [49]	Europe	IV	Orthopaedic and Trauma Examination
	Massey et al. [27]	North America	V	ResStudy Orthopaedic Examination
	Nieves‐Lopez et al. [33]	North America	V	Shoulder and Elbow Surgery
	Ozdag et al. [36]	North America	V	Orthopaedic In‐Training Examination
	Hayes et al. [17]	North America	V	Orthopaedic In‐Training Examination
	Lum [25]	North America	V	Orthopaedic In‐Training Examination
Quality control				
	Yüce et al. [50]	Europe	III	Rotator Cuff Injuries
Answering patients' frequently asked questions				
	Liu et al. [23]	North America	V	Total Knee Arthroplasty
	Gan et al. [14]	Asia	III	Total Knee Arthroplasty

### Decision making

#### Patient identification

In the context of surgical site infection (SSI) prevention in high‐risk paediatric spine surgery, Chester et al. evaluated ChatGPT‐4 for concordance with expert consensus throughout the care pathway [7]. Among 14 statements that reached expert agreement, ChatGPT was concordant with 13, but only exactly matched four. For nine statements that lacked expert consensus, ChatGPT4 aligned with expert responses in only three cases.

Zalikha et al. compared ChatGPT3.5 to a blinded orthopaedic surgeon in identifying a 1‐year minimum clinically important difference (MCID) for the Knee Osteoarthritis Outcome Score‐Joint Replacement (KOOS‐JR) from those who did not use electronic health records (EHRs) from 6 months preoperatively to 9–15 months postoperatively [51]. ChatGPT achieved 97% sensitivity, 33% specificity and 65% accuracy. The surgeon achieved 90%, 63% and 76%, respectively.

#### Surgical approach

Musbahi et al. compared ChatGPT3.5 and 73 orthopaedic surgeons in assessing 32 clinical cases to decide if total knee replacement (TKR) or unilateral knee replacement (UKR) was more appropriate [31]. Disagreement occurred in five (15.6%) scenarios. ChatGPT3.5 exhibited greater confidence for UKR (mean confidence = 2.4 vs. 1.7 for surgeons) and TKR (mean confidence = −2.0 vs. −1.1).

Liu et al. compared ChatGPT4 to eight orthopaedic surgeons in responding to five clinical emergency department scenarios regarding diagnosis, management, surgical indication and patient counselling [24]. Responses were evaluated using 5‐point Likert scale. AI demonstrated superior completeness (4.0 vs. 3.7, *p* = 0.001), helpfulness (4.2 vs. 3.7, *p* < 0.001), specificity (3.9 vs. 3.6, *p* = 0.001) and overall quality (4.0 vs. 3.8, *p* = 0.01), with no significant difference in accuracy (4.0 vs. 4.1, *p* = 0.61).

#### Radiograph reading

Zhu et al. evaluated ChatGPT‐4 on 117 anterior–posterior knee radiographs for osteoarthritis grading [53]. AI achieved 35% accuracy compared to over 80% for senior surgeons, with ChatGPT achieving 0.95 precision, 0.83 recall and F1 score 0.88 in binary classification.

### Management plans

In Dagher et al.'s study, ChatGPT‐4 was given 10 common orthopaedic scenarios, with its management plans compared to those made by orthopaedic attendings [9]. ChatGPT‐4's management plans aligned with the American Academy of Orthopaedic Surgeons (AAOS) guidelines in 90% of scenarios compared to 96% for physician‐created plans. Agreement between ChatGPT4 and physician‐generated plans was observed in 78% of cases.

McNamara et al. compared assessment and management plans generated by ChatGPT4 using real patient history, physical examination and imaging to the actual clinical course determined by the department's multispecialty trauma conference [28]. All plans were deemed clinically appropriate by the trauma conference panel of experts.

### Clinical knowledge

Yağar et al. assessed ChatGPT4 on the first step of the Turkish Orthopaedics and Traumatology Board Examinations between 2010 and 2023 [49]. ChatGPT's mean score was 70.2 compared to 58 for real examinees, with ChatGPT out‐competing across all years and sub‐specialities.

Massey et al. found ChatGPT3.5 scored 29.4%, ChatGPT4 scored 47.2% and orthopaedic residents scored 74.2% in 180 ResStudy Orthopaedic examination questions [27]. Residents significantly outperformed both LLMs (*p* < 0.001).

Nieves‐Lopez assessed ChatGPT3.5 and ChatGPT4 on 86 questions selected from the 2019 and 2021 AAOS shoulder‐elbow assessment questions [33]. The proficiency for medical education credit threshold is 50%. ChatGPT 3.5 and 4 answered 52.3% and 73.3% of the questions correctly, respectively (*p* = 0.003).

Ozdag et al. compared ChatGPT3.5 to orthopaedic residents in 102 upper‐extremity questions from the 2020–2022 Orthopaedic In‐Training Examination (OITE) [36]. Postgraduate Year 1 (PGY‐1) interns scored an average of 51%, compared to 45% for ChatGPT3.5, with PGY‐5 residents averaging 76%. Hayes et al. found ChatGPT4 scored 49% on 260 2019 OITE questions with PGY1‐5 scoring 53%–72% [17]. Lum found ChatGPT scored 47% on 207 questions OITE questions, placing it in the 40th percentile for PGY1s, eighth for PGY2s and first for PGY3‐5s [25].

### Quality control

Yüce et al. assessed ChatGPT4's ability to evaluate 40 YouTube videos on rotator cuff injuries using the DISCERN and RCSS evaluation framework [50]. The DISCERN tool is a standardised questionnaire designed to assess the quality of writing, while the RCSS evaluates the quality of diagnostic and surgical information for rotator cuff surgery [5]. Two human observers conducted the same assessments. ChatGPT4 generally assigned higher scores, with agreement between ChatGPT4 and human observers measured at AC1 = 0.575 (*p* = 0.025) for DISCERN and AC1 = 0.516 (*p* = 0.003) for RCSS. In 45% (18 of 40 videos), ChatGPT4 provided incorrect total RCSS scores.

### Answering patient FAQs

Liu et al. compared ChatGPT responses for 10 FAQs to five expert arthroplasty attendings [23]. Blinded evaluation using a 5‐point Likert scale found ChatGPT scored significantly higher than three attendings across empathy, accuracy, completeness and overall quality. When attending scores were averaged, ChatGPT scored higher for empathy (4.4 vs. 3.5), accuracy (4.4 vs. 3.7), completeness (4.4 vs. 3.5) and overall quality (4.4 vs. 3.6).

Gan et al. randomly assigned 60 patients to either Chat‐GPT‐assisted (where ChatGPT4 was used to provide responses to patient queries during the consent process, with surgeons interpreting and contextualising information) or surgeon‐led informed consent [14]. The ChatGPT group demonstrated significantly lower anxiety immediately after informed consent (HADS‐A: 10.48 vs. 12.75, *p* = 0.04; PAS‐7: 12.44 vs. 14.64, *p* = 0.01; VAS‐A: 5.40 vs. 6.71, *p* = 0.02) and on the fifth postoperative day (HADS‐A: 8.33 vs. 10.71, *p* = 0.01; VAS‐A: 3.41 vs. 4.64, *p* = 0.008). The ChatGPT group also reported higher satisfaction with preoperative education (4.22 vs. 3.43, *p* < .001) and overall hospitalisation experience (4.11 vs. 3.46, *p* = 0.001).

**Figure 2:**
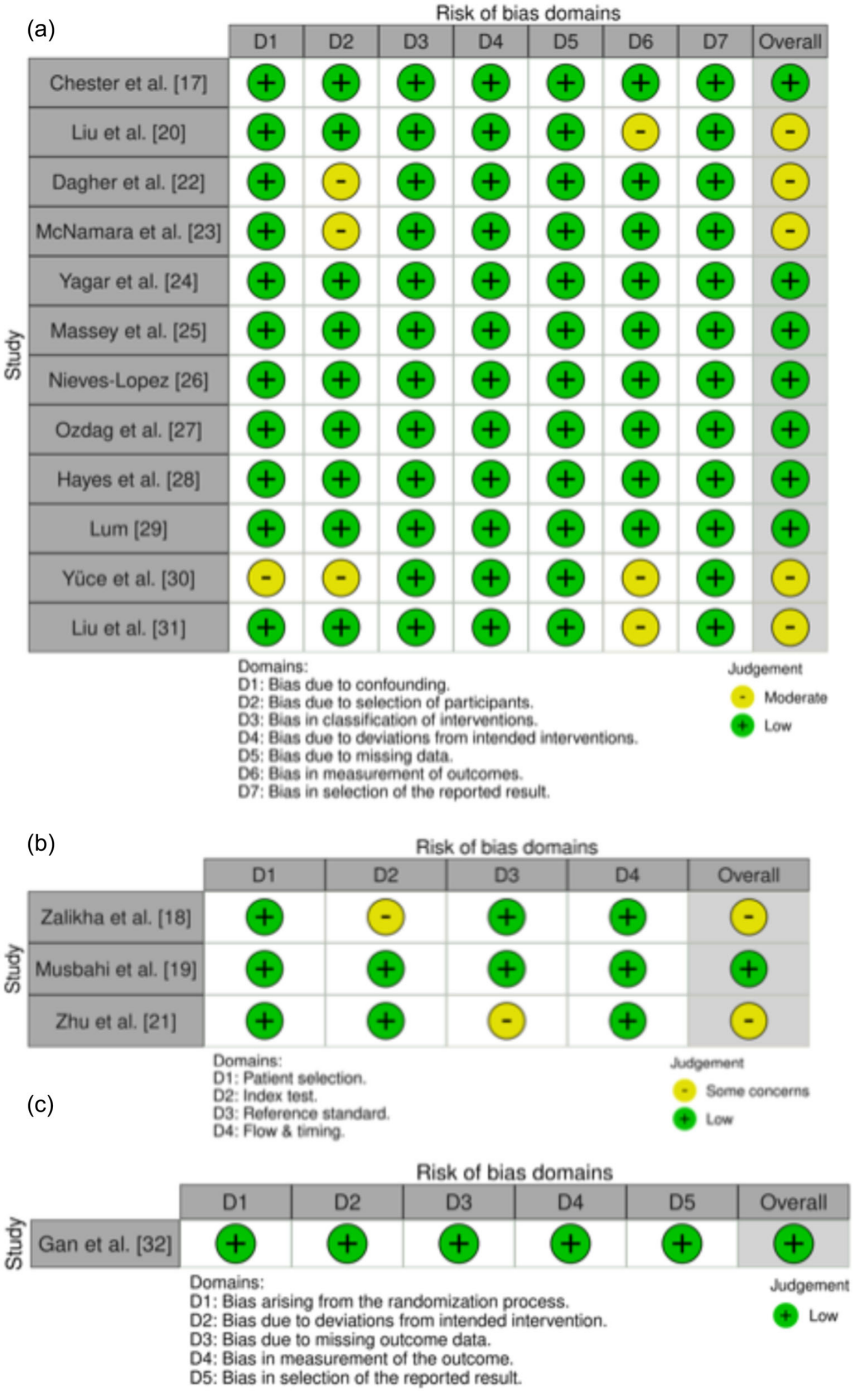
Risk of bias assessments, visualised using robvis: (a) ROBINS‐I for nonrandomised studies, (b) QUADAS‐2 for diagnostic accuracy studies and (c) RoB2.0 for randomised trials.

Risk of bias assessment using ROBINS‐I, QUADAS‐2 and Rob2.0 indicated that all studies were judged to be either low risk or moderate risk/some concerns (Figure 2).

## DISCUSSION

This study highlights that although AI demonstrates strengths in emergency trauma decision‐making and answering patient FAQs, AI was outperformed by surgeons and trainees in more complex tasks such as SSI risk prediction, MCID patient identification, arthritis radiographic interpretation and OITE performance.

Limitations with AI implementation are evident. ChatGPT3.5 demonstrated higher sensitivity but lower specificity and accuracy in identifying MCID for KOOS‐JR, over‐confidence in deciding between TKR and UKR and when assessing educational rotator cuff injury videos, potentially due to a lack of creativity and judgement [11, 31, 50, 51]. AI does not always align with clinician‐generated management plans, lacking the ability to differentiate acceptable management options, filter unreliable sources, perform independent assessments or learn from experience [1, 31, 32, 38]. AI remains limited to release‐date information, limiting responsiveness to new guidelines [19, 32]. Restricted access to paywalled content or non‐English content can miss critical publications and may introduce translational errors [35]. Hallucinations and fabricated references further undermine trust [2, 26, 35]. However, Deep Research models, trained to navigate the open web, aim to overcome reliance on training data and enable access to up‐to‐date research [52].

Gaps in orthopaedic AI research are highlighted in areas such as streamlining administration, an area 81% of orthopaedic surgeons view as beneficial and in supporting clinical research, where surgeon comparisons are limited [41]. For example, Brameier et al. used ChatGPT to create two randomised, controlled trial papers without actual data, one accepted and one referred to a second, illustrating the controversial role of AI in research and academic writing [2, 20]. Some journals require AI use to be acknowledged, whilst others, such as KSSTA, discourage it altogether [10]. AI may assist reference checking and summarising, though it cannot replace human expertise in contextualising, interpreting and refining outputs, though open‐access LLMs such as DeepSeek may improve study access [21]. ChatGPT has generated clinical letters for elective and fracture scenarios, readable at a 14–15‐year‐old level, and models exist for EHR data extraction [4, 34]. However, AI responses may reflect racial and sex biases from training data and incomplete EHR, common among underinsured or migrant patients, worsening socioeconomic healthcare disparities [3, 4, 8, 13].

Despite advances in algorithms, further research comparing AI outcomes to orthopaedic surgeons is critical to ensure safe and effective implementation that enhances patient care [6].

In other specialties, a systematic review by Takita et al. of 83 studies on AI for diagnostic tasks revealed diagnostic accuracy of 52.1%, with no significant difference compared to physicians overall (*p* = 0.10), although AI performed worse than experts (69.7%, *p* = 0.007) [45]. No differences were observed between general medicine and various specialities, including orthopaedics, except urology and dermatology, highlighting AI's broad capabilities but continued need for human judgement. There was a lower accuracy at 35% in osteoarthritis grading, highlighting challenges in more nuanced situations [53]. In general surgery, ChatGPT scored 39.6/50 for creating management plans, outperforming junior residents (33.4), but below senior residents (38.8, *p* = 0.009) [37]. This aligns with our results, indicating AI may support decision‐making but cannot replace experienced clinicians.

Clinicians report several limitations to the implementation of AI in clinical practice. While they may adopt AI to enhance efficiency, accuracy and productivity, they emphasise that tools must be intuitive and minimally disruptive [42]. Technical barriers like poor system design, lack of transparency, misalignment with clinical reasoning and data privacy risks. Clinicians also question AI conclusiveness, especially regarding evidence‐based recommendations. Overall, clinicians highlight that their involvement in the design, introduction and validation of AI systems is critical for adoption.

Future research may involve larger, prospective studies comparing AI performance to orthopaedic surgeons, as well as clinician‐alone versus clinician‐plus‐AI in real‐world clinical scenarios, including documentation and research support. Clinician involvement is essential to guide AI applications and minimise risks to patient care. Models must be regularly retrained to incorporate new guidance and demonstrate both safety and superiority to clinicians before routine implementation.

## LIMITATIONS

As AI continues to rapidly evolve, findings of this review may become outdated. Although 15 of the 16 included studies were published over the past three years, models evaluated, such as ChatGPT3.5 and ChatGPT4, may soon be replaced by emerging systems like Deep Think and Deep Research systems [44]. Furthermore, the scope of this study is limited as certain subspecialities such as paediatrics beyond spines and complex hand or upper limb traumas remain underexplored in current literature, limiting generalisability of findings. Methodological heterogeneity across studies further constrains the ability to draw definitive conclusions.

## CONCLUSION

AI can support orthopaedic care in answering patient FAQs and supporting emergency scenarios, though orthopaedic surgeons consistently outperform it in more complex tasks like risk factor identification and standardised examinations. Limitations including overconfidence, bias and reliance on outdated information prevent AI from replacing human expertise. Clinician involvement in design, validation and implementation is essential, with adoption into practice guided by rigorous studies to ensure safe and effective augmentation of clinician outputs.

## AUTHOR CONTRIBUTIONS

Jemima Russell, Jamie Rosen and Martinique Vella‐Baldacchino each made substantial contributions to this work, including study design, data collection, analysis and interpretation. Jemima Russell drafted the initial manuscript, and all authors (Jemima Russell, Jamie Rosen and Martinique Vella‐Baldacchino) were involved in revising the manuscript and gave final approval for the version to be published. All authors had access to all data in the study and took responsibility for the integrity of the data and the accuracy of the data analysis.

## CONFLICT OF INTEREST STATEMENT

The authors declare no conflicts of interest.

## ETHICS STATEMENT

The authors have nothing to report.

## Supporting information

Supplementary Table 1. Search terms searched into OVID Medline on August 13^th^ 2025.

## Data Availability

The data that support the findings of this study are openly available.
