# Supplementary figures and images for: Artificial intelligence fails to outperform orthopaedic surgeons: A systematic review
Source: J Exp Orthop. 2025 Nov 14;12(4):e70548. doi: 10.1002/jeo2.70548 (PMC12616488; doi:10.1002/jeo2.70548)

**Appendix**


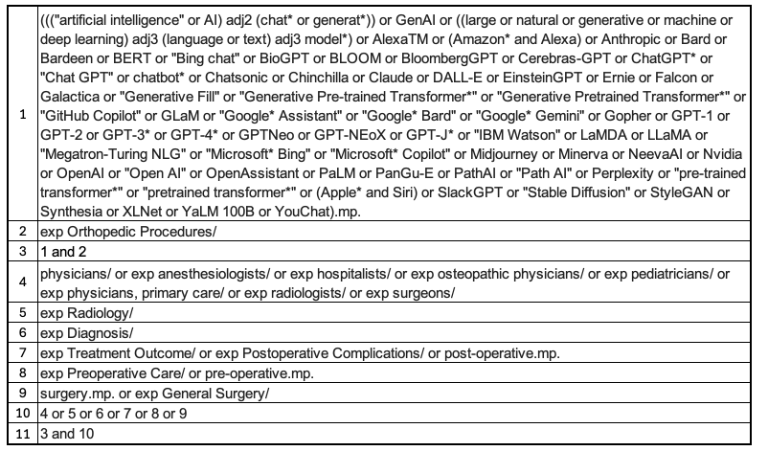


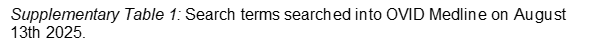

Supplement: Supplementary file 1 — Supplementary Table 1. Search terms searched into OVID Medline on August 13th 2025. [file JEO2-12-e70548-s001.docx]
